# Clinical Outcomes with Extended Depth of Focus Intraocular Lenses in Cases in Which Multifocal Lenses Are Not Primarily Recommended

**DOI:** 10.1155/2023/8814627

**Published:** 2023-06-16

**Authors:** María Teresa Álvarez-García, Carlota Fuente-García, Cristina Muñoz-Puyol, David P. Piñero

**Affiliations:** ^1^Vissum Miranza Madrid, Madrid, Spain; ^2^Department of Optics, Pharmacology and Anatomy, University of Alicante, Alicante, Spain

## Abstract

**Purpose:**

The purpose of the study is to evaluate the visual and patient-reported outcomes of patients undergoing cataract surgery with implantation of an extended depth of focus (EDOF) intraocular lens (IOL) who were not primarily good candidates for multifocal IOL implantation.

**Methods:**

Retrospective analysis of data from 30 eyes (23 patients) undergoing cataract surgery with implantation of one of two EDOF IOLs (follow-up: 37.9 ± 16.2 months) and prospective observational study including 106 eyes (78 patients) implanted with one of 6 different EDOF models (follow-up: 8.0 ± 7.7 months). Patients recruited had one of the following conditions: monofocal IOL implanted in the fellow eye, previous corneal refractive surgery, mild and nonprogressive maculopathy or glaucoma, age > 75 years, amblyopia, or previous vitrectomy.

**Results:**

In the retrospective phase, significant improvements were found in uncorrected distance (UDVA), corrected distance (CDVA), and corrected near visual acuity (CNVA) (*p* ≤ 0.013), with a nonsignificant trend to improvement in uncorrected near visual acuity (UNVA). A total of 90% of patients were completely to moderately satisfied with the outcome achieved. In the prospective phase, significant improvements were found in UDVA, CDVA, UNVA, and CNVA (*p* ≤ 0.032), with a total of 85.5% of patients being completely to moderately satisfied (dissatisfaction 3.3%). In both phases, extreme difficulties were only reported by a limited percentage of patients for performing some near vision activities.

**Conclusions:**

EDOF IOLs seem to be a viable option for providing an efficient visual rehabilitation with good levels of patient satisfaction and spectacle independence associated in patients that are not primarily good candidates for multifocal IOL implantation.

## 1. Introduction

The recent development of extended depth of focus (EDOF) intraocular lenses (IOLs) has led to a new concept in the management of presbyopia and refractive errors [1]. These implants have been designed with the aim of reducing dysphotopsias that are normally associated with refractive or diffractive multifocal IOLs, maintaining quality of vision and increasing the functional range of vision without correction [2–7]. Compared to trifocal diffractive IOLs, EDOF IOLs have shown to provide similar intermediate visual outcomes but significantly worse near visual outcomes [8–10]. Furthermore, EDOF IOLs have shown comparable visual quality outcomes at far compared to monofocal IOLs but achieving better near and intermediate visual results [11, 12].

The visual results with multifocal IOLs are dependent on factors affecting the patient's ability of neuroadaptation, such as the presence of additional ophthalmological conditions, age, or personality [13, 14]. There is a risk associated to this type of implants of maladaptation to dysphotopsic phenomena and reduced visual quality in low-contrast situations [15]. Specifically, patients who have ocular pathologic features, including glaucoma, age-related macular degeneration (ARMD), and epiretinal membrane (ERM), are not adequate candidates for multifocal implants [16]. Considering that EDOF IOLs are associated with less photic phenomena and less reduction of contrast sensitivity, [2–12] these implants could be an adequate option in such cases in which multifocal IOLs are not recommended due to high risk of postoperative visual complaints. Indeed, EDOF IOLs have demonstrated to induce a continuous range of focus, with minimal blurring areas between distance, intermediate and near foci, and consequently higher tolerance to residual refractive errors [17–19].

To this date, very few studies have been conducted to evaluate the potential of EDOF IOLs for providing a successful visual restoration in those cases which the indication of multifocal IOLs is risky and not fully accepted [20–22]. The aim of the current study was to evaluate the visual and patient-reported outcomes of patients undergoing cataract surgery with implantation of an EDOF IOL who were not good candidates for multifocal IOL implantation due to one of the following conditions (common exclusion criteria of studies evaluating the outcomes of trifocal diffractive IOLs): patients operated on with a monofocal IOL in the fellow eye, previous corneal refractive surgery with excimer laser, mild and nonprogressive maculopathy, mild and nonprogressive glaucoma, age over 75 years, amblyopia, or previous vitrectomy.

## 2. Methods

### 2.1. Design

This study was divided into two phases: an initial retrospective compilation of data from 30 eyes (23 patients) who had undergone cataract surgery with implantation of an EDOF IOL, and a prospective observational study including 106 eyes (78 patients) undergoing cataract surgery by phacoemulsification with EDOF IOL implantation conducted afterwards. Patients were recruited and operated on at Vissum Miranza clinic (Madrid, Spain). Both phases included patients suitable for cataract surgery but not being good candidates for multifocal IOLs due to their ophthalmological and/or clinical characteristics. In the retrospective phase, data were collected from patients operated on with cataract surgery between 2016 and 2018, whereas the prospective part was conducted between October 2018 and December 2020. All patients were informed about the nature of the study and signed an informed consent prior to their inclusion following the tenets of the Declaration of Helsinki. Furthermore, the study protocol was revised and approved by the Ethics Committee of Miranza.

### 2.2. Patients

Patients were eligible for inclusion in the study if they had one of the following conditions: operated on with a monofocal IOL in the fellow eye, previous corneal refractive surgery with excimer laser, mild and nonprogressive maculopathy (defined as exudates and/or retinal thickening within 2 disc diameters of the centre of the macula but outside 1 disc diameter, without clinically significant changes in the last 12 months), mild and nonprogressive glaucoma (defined as vertical Cup-to-Disk Ratio <0.65 and/or mild visual field defect not within 10 degrees of fixation characterized by a mean deviation better than −6 dB on Humphrey visual field 24-2, and without clinically significant changes in the last 12 months), amblyopia, age of more than 75 years old (higher possibility of developing pathological conditions over time and less scientific evidence of the efficacy and safety of multifocal IOLs in this type of population), and previous vitrectomy surgery. Patients were excluded if they had another ocular disease, such as corneal dystrophy, ocular inflammation, moderate-severe glaucoma, moderate-severe maculopathy, congenital ocular anomalies or capsular pseudoexfoliation syndrome, neurological disorders, and acute or chronic illness, when IOL implantation within the capsular bag was not possible (for example, damaged or insufficient capsular support), when patients were unable to provide informed consent (for example, vulnerable subjects), or when patients used medication potentially interfering with the results of the surgery or increasing its risks.

### 2.3. Intraocular Lenses

Patients were implanted with one of the following EDOF IOLs:

- Tecnis Symphony (Johnson & Johnson Vision Care, Inc., Santa Ana, CA, USA): one-piece, biconvex, and hydrophobic acrylic IOL, which is available in powers ranging from 5 to 34 D and has an intermediate addition of +1.75 D. The achromatic surface aims to correct the chromatic aberrations of the cornea, providing high contrast sensitivity. The design of this IOL combines a diffractive achromatic technology and negative spherical aberration correction to enlarge the depth of focus. This lens has an overall diameter of 13.0 mm, with a 6.0-mm optic. The lens received a CE mark in Europe in June 2014 and was the first EDOF-labeled IOL approved in the United States in 2016 [23].
- MiniWell Ready (SIFI MedTech, Catania, Italy): biconvex hydrophilic-hydrophobic copolymer pupil-dependent IOL, with three different optical zones that allow to increase the depth of focus. The inner zone induces positive spherical aberration, the middle zone induces negative spherical aberration, and the outer zone has a monofocal aspheric design. The transitions between the three optical zones are smooth and have a gradual change of power (active transition zones). The IOL is available in powers from 0 to 30 D. It has a total diameter of 10.75 mm, with a diameter of the optical zone of 6 mm. [24] Considering the potential pupil-dependence of this specific type of IOL, it was not implanted in the subgroups of patients older than 75 years old and those with glaucoma as the pupil size in these two subgroups could be very small, leading to a suboptimal outcome.
- Lucidis (Swiss Advanced Vision, SAV-IOL SA, Neuchâtel, Suiza): one-piece foldable, multizone, refractive, and aspherical hydrophilic acrylic IOL, with a 360° square edge design and closed-loop haptics. The lens has an optical diameter of 6.0 mm and an overall diameter of 10.8 mm or 12.4 mm, depending on power. The 1-mm aspheric zone occupies the center of the IOL and is surrounded by a 6-mm refractive ring. It is available in powers from 5 to 30 D, with an intermediate addition of +3 D. [25].
- Isopure 123 (PhysIOL sa/nv, Liège, Belgium): monofocal aspheric hydrophobic IOL, with a 360° square edge design and closed-loop haptics, providing enhanced intermediate vision. It is available in powers from 10 to 30 D, with an intermediate addition of +1.00 D. It incorporates the Isofocal technology, including a 100% monofocal refractive optic, combining an anterior/posterior surface profile of increased negative spherical aberration that is fine-tuned for each diopter on the whole optic. This IOL can have an optical diameter of 5.75 mm or 6 mm and an overall diameter of 10.75 mm or 11 mm, depending on the power [26].
- AcrySof IQ Vivity Extended Vision (Alcon Laboratories, Inc., Forth-Worth, TX, USA): single-piece hydrophobic acrylic IOL with an overall diameter of 13.0 mm and an optic zone of 6.0 mm of diameter. It is available in powers from 15 to 25 D, with an intermediate addition of 1.50 D. It uses a central 2.2-mm optical zone containing 2 nondiffractive transition elements that is beam shaped (X-Wave Technology), changing the wavefront of these central light beams to elongate the depth of focus. The anterior surface of the IOL is also designed with negative spherical aberration to compensate for the positive spherical aberration of the cornea [27].
- Precizon Presbyopic NVA (Ophthec BV, Groningen, The Netherlands): refractive IOL made of a hybrid material (Benz25) based on a hydrophobic acrylic coated with a hydrophobic surface. It has a C-loop shape, with an overall diameter of 12.5 mm and an optical zone of 6 mm of diameter. The design is based on the concept of “continuous transitional focus (CTF),” with a division of the optic into concentric sectors providing different levels of correction. The central sector, with a larger diameter, is dedicated to distance vision, whereas the two peripheral sectors are divided into multiple segments providing as a result a 60/40 ratio between near and far vision, with a smooth transition between distance, intermediate, and near vision. It has negative spherical aberration to compensate for the positive spherical aberration of the cornea and is available in powers from 1.0 to 35.0 D, with an addition of +2.75 D [5].

### 2.4. Clinical Protocol

In the prospective phase of the study, data collection was carried out in four visits, the first one prior to cataract surgery and another three visits after surgery (upon discharge from surgery, one year later, and at the last visit performed). All tests and evaluations were performed by the same group of professionals. On the first visit, all patients underwent a complete ocular and visual examination including anamnesis with personal data and family history, manifest refraction, uncorrected distance (UDVA) and near (measured at 40 cm) visual acuity (UNVA), corrected distance (CDVA) and near visual acuity (CNVA), slit lamp evaluation of both anterior and posterior segments of the eye, Goldmann applanation tonometry, corneal topography, pupillometry and aberrometry with the Sirius system (CSO, Florence, Italy), and macular analysis by optical coherence tomography with the Cirrus 5000HD (Cirrus HD-OCT 5000, Zeiss Meditec. Inc) and RTVue (Optovue RTVue XR Avanti, Optovue Inc., Fremont, CA) systems.

Postoperatively, the surgical discharge visit was carried out in all cases at 4–6 weeks after surgery, including the measurement of monocular UDVA, UNVA, CDVA, and CNVA, measurement of distance-corrected near visual acuity (DCNVA) and evaluation of the patient-reported outcomes in terms of near vision using the NAVQ-10 questionnaire, which is a Rasch-validated survey, allowing the assessment of patient satisfaction in near and intermediate vision activities and the level of spectacle independence [28]. At the one-year visit and at the last follow-up visit, the same examinations described at the surgical discharge visit were repeated.

In the retrospective phase, an attempt was made to collect all the information recorded in the electronic medical files of patients referring to the tests and examinations described above in each of the visits of the prospective phase. In addition, the information recorded in the last examination performed in each patient was also collected, which in some cases was performed up to 4 years after surgery. In addition, patients were contacted by phone to carry out the NAVQ10 satisfaction survey.

In all cases, optical biometry and IOL power calculations were performed before surgery with one of the following two devices of the same manufacturer: IOL-Master 700 and IOL-Master 500 (Carl Zeiss Meditec AG, Jena, Germany).

### 2.5. Statistical Analysis

Statistical analyses were performed with a commercially available software package (SPSS for Mac, Version 15.0; IBM Corporation, Armonk, NY, USA). Normality of data samples was evaluated by means of the Kolmogorov–Smirnov test. A descriptive analysis of the sample was performed by calculating the absolute and relative frequencies for the categorical variables, or with the mean, standard deviation, and range in the case of continuous variables. When parametric analysis was possible, the Student's *t* test for paired data was used for comparisons between consecutive visits, whereas the Wilcoxon ranked sum test was applied to assess the significance of such differences when parametric analysis was not possible. In the retrospective phase of the research, a comparison between the two IOL subgroups was performed using the Mann–Whitney test, as visual acuity data were not normally distributed in these two subgroups. Likewise, a multiple comparison between the different IOL subgroups in the prospective phase of the research was performed using the Kruskal–Wallis test, with the posthoc comparison by pairs with the Bonferroni-adjusted Wilcoxon test. Differences were considered as statistically significant when the associated *p* value was <0.05.

## 3. Results

### 3.1. Retrospective Study

The study was comprised of 11 men and 12 females with a mean age of 69.4 years (standard deviation, SD: 12.5; range: from 49 to 87 years). Data from a total of 17 right and 13 left eyes were included. Mean follow-up time was 37.9 months (SD: 16.2), ranging from 1 to 56 months. In this sample, half of the eyes were implanted with the Symfony IOL and the other half with the MiniWell IOL.  Table 1 summarizes the visual and refractive outcomes obtained in this sample of eyes. A statistically significant reduction was observed at the postoperative discharge visit in sphere and spherical equivalent (*p* < 0.001), as well as a significant improvement in UDVA (*p* < 0.001), CDVA (*p* < 0.001), and CNVA (*p*=0.013). There was a trend to improvement in UNVA that did not reach statistical significance (*p*=0.182).  Table 2 shows the CDVA and CNVA outcomes obtained in the retrospective phase of the current study according to the condition for which multifocal IOL implantation was not recommended: fellow eye implanted with monofocal IOL (10 eyes, 33.3%), previous vitrectomy (3 eyes, 10.0%), mild maculopathy (8 eyes, 26.7%), age of more than 75 years (10 eyes, 33.3%), and amblyopia (2 eyes, 6.7%).

**Figure 1:**
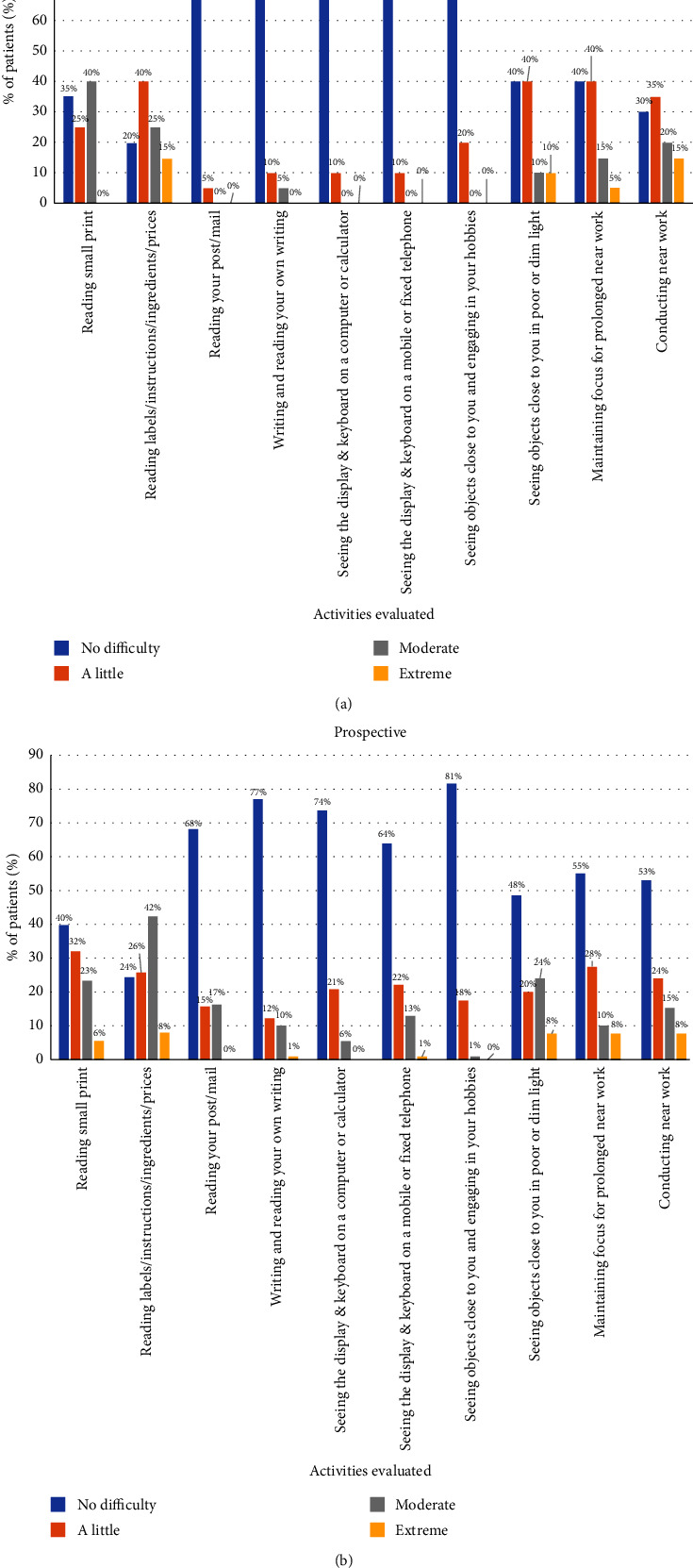
Distribution of postoperative difficulties in performing different near and intermediate visual activities evaluated with the NAVQ questionnaire in the retrospective (a) and prospective (b) phases of the current study.

The analysis of the patient-reported outcomes revealed that most of patients that answered the NAVQ questionnaire (20 patients) were satisfied with the vision achieved after surgery: completely satisfied (5 patients, 25.0%), very satisfied (7 patients, 35.0%), and moderately satisfied (6 patients, 30.0%). Only 2 patients (10.0%) referred to be dissatisfied with the visual outcome. Most patients referred no difficulty or a little difficulty after surgery in performing different near and intermediate visual activities, as shown in Figure 1. Only extreme difficulty was reported by a limited percentage of patients for performing the following activities: reading labels/instructions/ingredients/prices (15%), seeing close objects in poor or dim light (10%), maintaining focus for prolonged near work (5%), and conducting near work (15%) (Figure 1).

No significant differences in terms of preoperative and postoperative UDVA, CDVA, UNVA, and CNVA were found between eyes implanted with the Symfony IOL and those implanted with the MiniWell IOL (*p* ≥ 0.176), except for UNVA at the postoperative discharge visit (Symfony 0.18 ± 0.11 vs. MiniWell 0.31 ± 0.16, *p*=0.043). A total of 93.3% and 100% of eyes implanted with the MiniWell and Symfony IOL, respectively, had a spherical equivalent within ±0.50 D at the postoperative discharge visit (*p*=0.309). Concerning YAG capsulotomy, it was needed during the follow-up in a total of 6 eyes (20.0%).

### 3.2. Prospective Study

In this phase of the study, a total of 106 eyes (41 male and 37 females) with a mean age of 67.6 years (standard deviation, SD: 10.2; range: from 36 to 88 years) were enrolled. A total of 51 right and 55 left eyes were operated on. Mean follow-up time was 8.0 months (SD: 7.7), ranging from 1 to 32 months. The distribution of the IOLs implanted was as follows: Isopure (7 patients, 6.6%), Lucidis (11 patients, 10.4%), MiniWell (1 patient, 0.9%), Precizon (13 patients, 12.3%), Symfony (36 patients, 34.0%), and Vivity (38 patients, 35.9%). Due to the limited number of patients implanted with some specific models of these EDOF IOLs, no statistical analysis of the comparison between them was performed as it would be biased.  Table 3 summarizes the visual and refractive outcomes obtained in this prospective phase of the research. Besides a significant change in refraction (*p* < 0.001), significant improvements were found in UDVA (*p* < 0.001), CDVA (*p* < 0.001), UNVA (*p*=0.032), and CNVA (*p*=0.003) at the postoperative discharge visit.  Table 4 summarizes the preoperative and postoperative CDVA and CNVA data according to the condition for which multifocal IOL implantation was not recommended: fellow eye implanted with monofocal IOL (32 eyes, 30.2%), previous vitrectomy (15 eyes, 14.2%), mild maculopathy (26 eyes, 24.5%), age of more than 75 years (24 eyes, 22.6%), amblyopia (2 eyes, 1.9%), incipient glaucoma (4 eyes, 3.8%), and previous refractive surgery (17 eyes, 16.0%).

In this prospective phase of the research, the NAVQ questionnaire was answered by most of patients (90 patients, 84.9%), reporting most of them that were satisfied with the vision achieved after surgery: completely satisfied (20 patients, 22.2%), very satisfied (35 patients, 38.9%), moderately satisfied (22 patients, 24.4%), and a little satisfied (10 eyes, 11.1%). Only 3 patients (3.3%) referred to be dissatisfied with the visual outcome. Most patients referred no difficulty or a little difficulty after surgery in performing different near and intermediate visual activities, as shown in Figure 1, with more cases of moderate difficulty reported for those activities involving near distances. Only extreme difficulty was reported by a limited percentage of patients for performing the following activities: reading small print (6%), reading labels/instructions/ingredients/prices (8%), writing and editing their own writing (1%), seeing the display and keyboard on a computer or calculator (1%), seeing close objects in poor or dim light (8%), maintaining focus for prolonged near work (8%), and conducting near work (8%) (Figure 1).

Concerning YAG capsulotomy, it was needed during the follow-up in a total of 9 eyes (8.5%).

## 4. Discussion

The implantation of multifocal IOLs has increased significantly in recent years since patients increasingly demand a complete solution for presbyopia leading to spectacle independence in their daily lives [13]. These IOLs provide adequate vision at far, intermediate, and near distances [13]. However, most multifocal IOL designs split light and generate multiple retinal foci, being the final outcome very sensitive to several factors such as IOL centration, transparency of the posterior capsule, the significant presence of high-order aberrations in the eye, tear film stability, and the pupil size or retinal or neurological factors complicating the neuroadaptation to this pattern of multiple foci [15]. For this reason, the presence of ocular pathologies that can affect over time to the patient's visual capabilities is considered as a contraindication for the implantation of multifocal IOLs [16]. Other conditions such as patients operated on with a monofocal IOL in the fellow eye, previous corneal refractive surgery with excimer laser, age over 75 years, or amblyopia have been also suggested to be relative contraindications to multifocality as the implantation of a multifocal IOL can compromise the patient's visual quality due to more difficulty in the neuroadaptation ability or a baseline already compromised visual function [29]. Alternatives such as monovision or EDOF IOLs [20–22] have been described for this typology of patients. It should be considered that EDOF IOLs were designed with the aim of reducing dysphotopsia, with less reduction in contrast sensitivity [2–12] and higher tolerance to residual refractive errors than multifocal IOLs [17–19]. In the current series, we evaluated retrospectively and prospectively the clinical and patient-reported outcomes of cataract surgery with implantation of EDOF IOLs in patients with profiles being relative contraindications to multifocal IOL implantation.

The research was divided into two phases: one retrospective analysis of already available data in our clinical setting and a prospective study. In these two phases, a great variety of EDOF IOLs were used, including a total of 6 different types of commercially available EDOF IOLs: Tecnis Symphony, MiniWell, Lucidis, Vivity, Isopure, and Precizon Presbyopic NVA. The main optical principle of these IOL models is to create an elongated focal point allowing an acceptable level of image quality for a range of distances from far to intermediate-near vision. In general, in both prospective and retrospective phases, a significant improvement was found in UDVA, CDVA, and CNVA, confirming the ability of this type of IOLs of providing a successful visual restoration in those cases considered in the study for who multifocal IOLs were contraindicated. This was consistent with the good patient-reported outcomes obtained, with most of the patients from the retrospective and prospective samples referring to be completely satisfied, very satisfied, or moderately satisfied with the visual outcome obtained after surgery.

In the retrospective phase of the study, the results were evaluated in a sample of 30 eyes from 23 patients with a long-term follow-up (mean follow-up: 37.9 ± 16.2 months) that were implanted with one of the following two EDOF IOLS: Symfony or MiniWell. Mean CDVA changed significantly from a preoperative value of 0.22 ± 0.18 logMAR to a mean value of 0.08 ± 0.08 logMAR at the postoperative discharge visit, without significant variations at 1 year after surgery and at the last postoperative visit. A similar trend was observed for UDVA, with a significant improvement at the postoperative discharge visit (from 0.68 ± 0.46 to 0.18 ± 0.19 logMAR) and a maintenance of the visual gain obtained afterwards. There was also a trend to improvement in UNVA, but the change did not reach statistical significance (from 0.45 ± 0.41 to 0.26 ± 0.16). Furthermore, a comparative analysis was performed between the two EDOF IOLs implanted, finding a significantly better UNVA with the Symfony IOL compared to MiniWell. This is consistent with a previous research showing significant differences in the level of depth of field achieved with these two types of IOLs in a not compromised population, with higher values of depth of focus measured with the iTrace system with the Symfony IOL [30]. Despite this visual difference between IOLs, nonsignificantly different levels of predictability (100% vs. 93.3% with spherical equivalent within ±0.50 D) and patient's satisfaction were found. Regarding the difficulty in performing different daily activities, a limited portion of patients referred extreme difficulties and always in reference to near activities (15% reading labels/instructions/ingredients/prices, 10% seeing close objects in poor or dim light, 5% maintaining focus for prolonged near work, and 15% conducting near work). In general, it can be concluded according to the data obtained in the questionnaire that the level of visual performance without spectacles was better for intermediate than for near vision, as could be expected considering the optical basis of EDOF IOLs [17].

Considering these first results obtained in the retrospective phase, we designed and conducted a prospective study, but including more models of EDOF IOLs. Specifically, a total of 106 eyes of 78 patients were enrolled, with a mean follow-up of 8.0 ± 7.7 months. In this sample, improvements in both UDVA (from 0.65 ± 0.41 to 0.15 ± 0.17) and UNVA (from 0.40 ± 0.37 to 0.24 ± 0.19) at the postoperative discharge visit were statistically significant, with also a significant gain in CDVA. As happened in the retrospective phase, these improvements were maintained during the remaining follow-up. Regarding patient's satisfaction, half of the sample referred to be completely satisfied or very satisfied with respect to performance without glasses in intermediate and near vision tasks, which is a percentage somewhat lower than that obtained in the retrospective study (60%), but only 11.5% expressed little satisfaction or completely dissatisfied (very similar to the retrospective study, where it was 10%). No statistically significant differences were found between the implanted IOL model and the degree of patient's satisfaction. Furthermore, as evidenced in the retrospective study survey, the visual performance without spectacles was higher for intermediate vision tasks than for near vision tasks, but ranging from 100% to 70–80%. Between 40 and 70% of near vision tasks could be performed comfortably without glasses, percentages slightly lower than those obtained in the retrospective study. Extreme difficulty was reported mainly for near vision activities but in percentages of 8% or below.

In the subgroup of patients with previous refractive surgery, the visual outcomes were also excellent, with no limitation in terms of refractive predictability, as other authors have also reported with EDOF IOLs [31–33]. Palomino-Bautista et al. [31] evaluated the predictability of the symphony IOL 3 months after being implanted in 76 eyes of 43 patients with previous LASIK, obtaining a total of 62.6% with a postoperative spherical equivalent within ±0.5 D and 86.3% within ±1 D, which is consistent with our results. Another study published by Ferreira et al. [33] compared the results obtained with a monofocal IOL (Tecnis ZCB00) with those obtained with an EDOF IOL (Symphony) at 3 months after being implanted in patients with previous LASIK. These authors did not find significant differences in terms of refraction or contrast sensitivity between IOLs, but the binocular visual acuity without optical correction in intermediate and near vision was significantly better in the group of patients implanted with the EDOF IOL [33].

Regarding the use of EDOF IOLs in patients with glaucoma, Ouchi and Kinoshita [34] published a prospective study in 2015 including 15 eyes with different pathologies, including glaucoma, that underwent cataract surgery with implantation of a multifocal IOL. They reported postoperative contrast sensitivity data similar to that corresponding to age-matched healthy subjects, with none of them reporting poor visual quality and 80% of patients that could manage without the need of spectacles for near vision. In our series, the sample of patients with glaucoma was small (*n* = 4), although all of them showed good visual outcomes and predictability. This should be investigated further with larger sample size ton extract consistent conclusions.

Patients with age-related macular degeneration (ARMD) or epiretinal membrane (ERM) are generally not candidates for a multifocal IOL [29, 35]. In these cases, there is a risk of loss of contrast sensitivity, metamorphopsia, and development of postsurgical macular oedema that can reduce visual acuity and less refractive predictability [36, 37]. In our prospective study, we have evaluated a total of 41 eyes affected by retinal pathology, 15 of them with previous vitrectomy due to several reasons (mainly to retinal detachment and ERM), and 26 with mild and nonprogressive maculopathies, which included maculopathies associated with age and ERM that would have been stable over time. Once again, all of them presented good visual outcomes and predictability, with high levels of predictability associated, as in the subgroup of patients over 75 years of age, with amblyopia, and with monofocal IOL implanted in the fellow eye.

Concerning amblyopia, our sample was very limited to extract consistent conclusions, but the preliminary data of the small group of patients evaluated showed acceptable results. To our knowledge, there are no previous reports showing the results of EDOF IOL implantation in this condition. Only some experiences of multifocal IOL implantation have been reported, with also acceptable results obtained and no unwanted side effects [38].

Regarding the level of patient's satisfaction, there are few publications that evaluate the satisfaction of patients with EDOF IOLs in the presence of ophthalmological comorbidities. Baartman et al. [39] reported in a retrospective study evaluating radial keratotomy patients implanted with the Symfony IOL that 78% of them were satisfied with their vision after surgery and that a 44% were able to perform all their activities without glasses. In our prospective study, 50% of patients reported being satisfied or very satisfied with the visual result, being able to perform between 70 and 80% of the intermediate vision tasks without glasses and between 40 and 70% of the near vision tasks vision without glasses. The results of our study are inferior to those of other publications of EDOF IOLs implanted in patients without ophthalmological comorbidities [11, 40, 41] and are more in line with the outcomes from Baartman et al. [39]. In our retrospective study, 60% of patients reported being satisfied or very satisfied with the visual result and could comfortably perform between 60 and 80% of near vision activities without glasses.

This investigation has several limitations that should be acknowledged. First, the number of eyes implanted with some models of EDOF IOLs was limited and it must be increased to extract more consistent conclusions about differences in the sample of eyes evaluated between types of IOL and also between conditions. Second, DCNVA was only evaluated postoperatively in the prospective study, which is a parameter not biased by the residual refractive error. Future studies must include the analysis of this parameter as one of the main outcome measures. Third, intermediate visual acuity and contrast sensitivity were not evaluated and this is also a pending aspect to investigate in future series. However, despite these limitations, to our knowledge, this is the first study conducted to evaluate retrospectively and prospectively the viability of EDOF IOL implantation in medium and long term in 7 clinical situations that are usually considered contraindications to multifocal IOL implantation.

In conclusion, cataract surgery with implantation of an EDOF IOL is a useful option for providing an efficient visual rehabilitation with good levels of patient satisfaction and spectacle independence associated in eyes with some conditions that are normally considered as contraindications to multifocal IOL implantation, including patients operated on with a monofocal IOL in the fellow eye, previous corneal refractive surgery with excimer laser, mild and nonprogressive maculopathy, age over 75 years, and/or previous vitrectomy. This potential effective visual rehabilitation has been also observed in mild and nonprogressive glaucoma and amblyopia, but futures studies with larger samples are needed to confirm these preliminary outcomes. Therefore, EDOF IOLs seems to be viable option that may be considered as an alternative to monofocal IOLs in this type of patients.

## Figures and Tables

**Table 1:** Visual and refractive outcomes obtained in the retrospective phase of the current study. Abbreviations: SD, standard deviation; UDVA, uncorrected distance visual acuity; CDVA, corrected visual acuity; UNVA, uncorrected near visual acuity; CNVA, corrected near visual acuity.

	Preoperative		Postoperative discharge visit			Postoperative 1 year		Postoperative last visit		
	*N*	Mean (SD)	*N*	Mean (SD)	*p*-value	*N*	Mean (SD)	*N*	Mean (SD)	*p*-value
		Range		Range			Range		Range	
Sphere (D)	30	−1.62 (2.87)	30	−0.10 (0.62)	<0.001	22	−0.07 (0.77)	20	−0.09 (0.83)	<0.001
		−7.00 to 2.75		−2.50 to 1.00			−2.75 to 1.00		−3.75 to 1.25	

Cylinder (D)	30	−0.92 (0.73)	30	−0.51 (0.39)	0.188	22	−0.56 (0.41)	20	−0.74 (0.26)	0.382
		−2.75 to 0.00		−1.25 to 0.00			−1.25 to 0.00		−1.25 to −0.50	

Spherical equivalent (D)	30	−2.06 (2.78)	30	−0.35 (0.66)	<0.001	22	−0.32 (0.79)	20	−0.34 (0.84)	<0.001
		−7.50 to 2.12		−3.12 to 0.75			−3.25 to 0.75		−4.00 to 1.00	

LogMAR UDVA	26	0.68 (0.46)	30	0.18 (0.19)	<0.001	23	0.19 (0.27)	30	0.19 (0.20)	<0.001
		0.10 to 1.70		0.00 to 1.00			−0.08 to 1.30		0.00 to 1.00	

LogMAR CDVA	30	0.22 (0.18)	30	0.08 (0.08)	<0.001	23	0.09 (0.06)	28	0.07 (0.10)	<0.001
		0.01 to 0.70		0.00 to 0.32			−0.08 to 0.40		−0.06 to 0.40	

LogMAR UNVA	11	0.45 (0.41)	21	0.26 (0.16)	0.182	18	0.28 (0.17)	22	0.29 (0.16)	0.247
		0.00 to 1.40		0.10 to 0.70			0.00 to 0.70		0.01 to 0.70	

LogMAR CNVA	23	0.19 (0.17)	26	0.09 (0.07)	0.013	21	0.06 (0.07)	28	0.08 (0.09)	0.010
		0.00 to 0.52		0.00 to 0.22			0.00 to 0.30		0.00 to 0.30	

**Table 2:** Visual outcomes obtained in the retrospective phase of the current study according to the condition for which multifocal IOL implantation was not recommended. Abbreviations: SD, standard deviation; IOL, intraocular lens; CDVA, corrected visual acuity; CNVA, corrected near visual acuity.

	Condition	Preoperative		Postoperative discharge visit		
		*N*	Mean (SD)	*N*	Mean (SD)	*p -*value
			Range		Range	
LogMAR CDVA	Fellow eye with monofocal IOL	10	0.30 (0.22)	10	0.05 (0.10)	<0.001
			0.05 to 0.70		0.00 to 0.32	
	Previous vitrectomy	3	0.24 (0.19)	3	0.06 (0.04)	^*∗*^
			0.03 to 0.40		0.03 to 0.10	
	Mild maculopathy	8	0.18 (0.16)	8	0.09 (0.07)	0.185
			0.03 to 0.52		0.03 to 0.22	
	Age > 75 years	10	0.30 (0.16)	10	0.09 (0.10)	0.002
			0.05 to 0.52		0.00 to 0.32	
	Amblyopia	2	0.22 (0.00)	2	0.22 (0.00)	^*∗*^
			0.22 to 0.22		0.22 to 0.22	

LogMAR CNVA	Fellow eye with monofocal IOL	7	0.22 (0.18)	8	0.04 (0.05)	0.040
			0.10 to 0.52		0.00 to 0.10	
	Previous vitrectomy	1	0.40 (0.00)	3	0.03 (0.06)	^*∗*^
			0.40 to 0.40		0.00 to 0.10	
	Mild maculopathy	7	0.24 (0.17)	7	0.13 (0.06)	0.053
			0.10 to 0.52		0.10 to 0.22	
	Age > 75 years	8	0.27 (0.18)	8	0.11 (0.04)	0.020
			0.10 to 0.52		0.10 to 0.22	
	Amblyopia	2	0.22 (0.00)	2	0.22 (0.00)	^*∗*^
			0.22 to 0.22		0.22 to 0.22	

^*∗*^Not enough sample size to provide statistical analysis.

**Table 3:** Visual and refractive outcomes obtained in the prospective phase of the current study. Abbreviations: SD, standard deviation; UDVA, uncorrected distance visual acuity; CDVA, corrected visual acuity; UNVA, uncorrected near visual acuity; CNVA, corrected near visual acuity; DCNVA, distance-corrected near visual acuity.

	Preoperative		Postoperative discharge visit			Postoperative 1 year		Postoperative last visit		
	*N*	Mean (SD)	*N*	Mean (SD)	*p -*value	*N*	Mean (SD)	*N*	Mean (SD)	*p -*value
		Range		Range			Range		Range	
Sphere (D)	106	−1.62 (3.64)	105	−0.02 (0.41)	<0.001	106	−0.02 (0.40)	104	−0.03 (0.39)	<0.001
		−14.00 to 3.75		−1.00 to 1.00			−1.00 to 0.75		−1.00 to 1.00	

Cylinder (D)	106	−1.20 (0.91)	105	−0.62 (0.51)	<0.001	106	−0.52 (0.46)	104	−0.75 (0.36)	<0.001
		−4.00 to 0.00		−2.00 to 0.00			−1.75 to 0.00		−1.75 to 0.00	

Spherical equivalent (D)	106	−2.16 (3.67)	105	−0.28 (0.38)	<0.001	106	−0.11 (0.30)	104	−0.26 (0.37)	<0.001
		−15.00 to 3.25		−1.25 to 0.62			−1.25 to 0.50		−1.25 to 0.50	

LogMAR UDVA	90	0.65 (0.41)	106	0.15 (0.17)	<0.001	46	0.13 (0.15)	104	0.14 (0.17)	<0.001
		0.01 to 1.70		−0.04 to 1.00			0.00 to 0.62		0.00 to 1.00	

LogMAR CDVA	106	0.19 (0.22)	106	0.08 (0.14)	<0.001	46	0.05 (0.07)	104	0.07 (0.14)	<0.001
		−0.08 to 1.30		0.00 to 1.00			−0.08 to 0.22		0.00 to 1.00	

LogMAR UNVA	29	0.40 (0.37)	90	0.24 (0.19)	0.032	41	0.24 (0.14)	94	0.23 (0.19)	0.023
		0.00 to 1.40		0.00 to 1.00			0.00 to 0.70		0.00 to 1.00	

LogMAR CNVA	100	0.15 (0.16)	102	0.09 (0.13)	0.003	46	0.08 (0.09)	102	0.08 (0.13)	0.002
		0.00 to 0.70		0.00 to 1.00			0.00 to 0.40		0.00 to 1.00	

LogMAR DCNVA	—	—	52	0.27 (0.21)	—	13	0.23 (0.18)	57	0.25 (0.22)	—
				0.00 to 1.00			0.00 to 0.70		0.00 to 1.00	

**Table 4:** Visual outcomes obtained in the prospective phase of the current study according to the condition for which multifocal IOL implantation was not recommended. Abbreviations: SD, standard deviation; IOL, intraocular lens; CDVA, corrected visual acuity; CNVA, corrected near visual acuity.

	Condition	Preoperative		Postoperative discharge visit		
		*N*	Mean (SD)	*N*	Mean (SD)	*p -*value
			Range		Range	
LogMAR CDVA	Fellow eye with monofocal IOL	32	0.20 (0.17)	32	0.06 (0.09)	<0.001
			0.00 to 0.70		0.00 to 0.47	
	Previous vitrectomy	15	0.28 (0.32)	15	0.05 (0.07)	<0.001
			0.00 to 1.30		0.00 to 0.22	
	Mild maculopathy	26	0.23 (0.34)	26	0.14 (0.24)	0.080
			0.00 to 1.30		0.00 to 1.00	
	Age > 75 years	24	0.18 (0.17)	24	0.09 (0.16)	0.022
			0.00 to 0.70		0.00 to 0.70	
	Amblyopia	2	0.41 (0.02)	2	0.21 (0.27)	^*∗*^
			0.40 to 0.42		0.02 to 0.40	
	Incipient glaucoma	4	0.01 (0.01)	4	0.01 (0.02)	^*∗*^
			0.00 to 0.02		0.00 to 0.05	
	Previous refractive surgery	17	0.13 (0.13)	17	0.07 (0.08)	0.166
			−0.08 to 0.47		0.00 to 0.22	

LogMAR CNVA	Fellow eye with monofocal IOL	32	0.19 (0.18)	31	0.09 (0.07)	<0.001
			0.00 to 0.70		0.00 to 0.40	
	Previous vitrectomy	13	0.15 (0.17)	14	0.07 (0.08)	0.046
			0.00 to 0.52		0.00 to 0.22	
	Mild maculopathy	24	0.11 (0.16)	25	0.13 (0.21)	0.253
			0.00 to 0.70		0.00 to 1.00	
	Age > 75 years	24	0.17 (0.17)	23	0.08 (0.10)	0.028
			0.00 to 0.70		0.00 to 0.40	
	Amblyopia	1	0.00 (0.00)	2	0.20 (0.28)	^*∗*^
			0.00 to 0.00		0.00 to 0.40	
	Incipient glaucoma	4	0.05 (0.06)	4	0.02 (0.05)	^*∗*^
			0.00 to 0.10		0.00 to 0.10	
	Previous refractive surgery	15	0.11 (0.13)	17	0.06 (0.06)	0.134
			0.00 to 0.40		0.00 to 0.22	

^*∗*^Not enough sample size to provide statistical analysis.

## Data Availability

The data used to support the study are available on a reasonable request.
